# Neck Swelling in a Critically Ill Patient With COVID-19-Related Acute Respiratory Distress Syndrome on Venovenous Extracorporeal Membrane Oxygenation: Consider the Differential

**DOI:** 10.7759/cureus.30877

**Published:** 2022-10-30

**Authors:** Eric Sy, Sherma Zacharias, James S Lee

**Affiliations:** 1 Department of Critical Care, Saskatchewan Health Authority, Regina, CAN

**Keywords:** covid-19 and cardiovascular complications, covid-19, subacute thyroiditis, clinical case report, severe acute respiratory distress syndrome, venovenous extracorporeal membrane oxygenation

## Abstract

Neck swelling during venovenous extracorporeal membrane oxygenation (VV-ECMO) usually heralds the development of potentially serious complications, including superior vena cava (SVC) syndrome, hematoma, and/or angioedema. In this case report, we describe a 43-year-old male patient who had received VV-ECMO support for the coronavirus disease 2019 (COVID-19) acute respiratory distress syndrome. During his hospitalization, he developed acute onset of neck swelling after two weeks of VV-ECMO and two days after a tracheostomy. Clinical examination and investigations were performed to exclude ECMO-related SVC syndrome and tracheostomy-related complications. Consequently, it was discovered the patient had developed COVID-19-related subacute thyroiditis with enlargement of both thyroid glands. Conservative management, including the use of continued glucocorticoids, raising the head of the bed, and observing for complications of thyroiditis, was undertaken. Eventually, this patient’s neck swelling resolved on its own, and he was eventually decannulated from ECMO several weeks later. Our case report highlights the differential diagnosis of neck swelling during VV-ECMO and considers the evaluation of different etiologies.

## Introduction

The development of neck swelling and facial edema during venovenous extracorporeal membrane oxygenation (VV-ECMO) usually heralds potentially serious complications in pediatric and adult ECMO, such as superior vena cava (SVC) obstruction [1,2]. Improper cannula sizing could lead to partial or complete venous obstruction. In pediatric patients, SVC flow obstruction from large ECMO cannulae may occur due to mechanical obstruction in the setting of a narrow blood vessel, but it has rarely been described in adults, most likely due to larger blood vessels in this population [1,2]. For most adult patients, the typical internal jugular vein ECMO cannula size may range from 15-32 French (Fr) depending on the cannula and ECMO configuration (i.e., single versus dual-lumen cannula). Additionally, ECMO cannula selection in Fr sizing could be aided with ultrasonography by measuring the diameter (D) of the target blood vessel and using the formula (Fr = D (in mm) × 3) [3]. However, there have been some isolated case reports in adults of SVC syndrome occurring in patients with both single-lumen and double-lumen catheters [1,2].

In critically ill patients, facial edema and acute neck swelling may be the result of other common complications, such as angioedema, anaphylaxis, and hematoma, or even as the consequence of prone positioning [4]. In this report, we describe the case of a 43-year-old man with coronavirus disease 2019 (COVID-19) acute respiratory distress syndrome (ARDS) who developed COVID-19-related subacute thyroiditis during VV-ECMO, manifesting with acute neck swelling.

## Case presentation

A 43-year-old man presented to our hospital with a one-day history of shortness of breath and several days of diarrhea, dry cough, and weakness. Five days prior to arrival, he was diagnosed to have COVID-19 pneumonia with the severe acute respiratory syndrome coronavirus 2 (SARS-CoV-2) variant B.1.1.7 (alpha variant). He was not vaccinated against COVID-19. He was subsequently admitted and placed on high-flow nasal oxygen. His medical history included type 2 diabetes, fatty liver disease, and nephrolithiasis. He had no personal or family history of autoimmune disease, thyroid disease, angioedema, and/or neck swelling. Social history included a remote history of smoking with a total of five pack-years. On examination, he was alert and oriented. His heart rate was 80 beats per minute, blood pressure was 117/64 mmHg, respiratory rate was 20 breaths per minute, oxygen saturation was 96% on 100% high-flow nasal oxygen, and temperature was 36.7°C. His weight was 89.2 kg and his height was 178 cm. He had bilateral bibasilar crackles with increased work of breathing. The remainder of his physical examination was normal.

He had received dexamethasone 6 mg orally daily since admission, but his clinical condition had deteriorated by hospital day eight. He was admitted to the intensive care unit and received four hours of non-invasive ventilation. Despite non-invasive ventilation, he continued to worsen. He subsequently was placed on invasive mechanical ventilation and received prone positioning for a ratio of the partial pressure of arterial oxygen to the fraction of inspired oxygen (PaO_2_:FiO_2_) of 73 mmHg. His condition deteriorated further, and he was cannulated onto VV-ECMO support using a 21 Fr Bio-Medicus return cannula (Medtronic, United States) in his right internal jugular vein and a 29 Fr Bio-Medicus drainage cannula in his right femoral vein on hospital day 11 for persistent mixed hypoxemic hypercarbic respiratory failure despite optimal ventilator settings. VV-ECMO blood flow was set at approximately 5.1 L/minute and the patient was anticoagulated with heparin. Additional lines included a left subclavian triple lumen 7 Fr central line, orogastric tube, and esophageal balloon (Cooper Surgical, United States). A tracheostomy was performed on ECMO day 13 (hospital day 24) using a Shiley 7CN80H tracheostomy tube (Medtronic, United States) with a percutaneous dilation technique.

**Figure 1:**
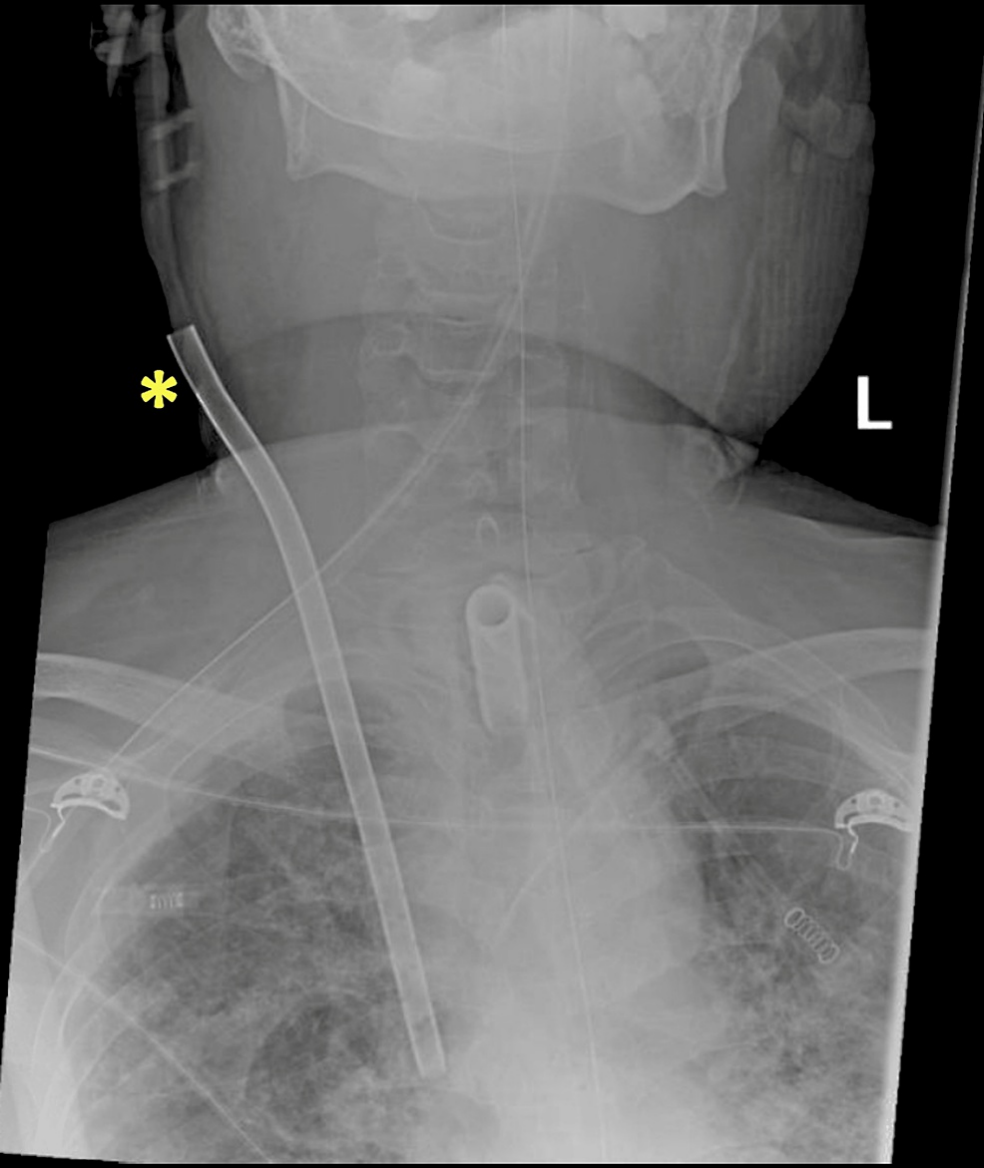
Anteroposterior radiograph of the head, neck, and chest. The yellow asterisk denotes the extracorporeal membrane oxygenation catheter.

Unfortunately, the patient developed marked neck swelling by ECMO day 15 (hospital day 26). Vital signs at the time included a heart rate of 73 beats per minute, blood pressure of 137/71 mmHg on 0.04 µg/kg/minute of norepinephrine, temperature of 37.3°C, and his settings on the ventilator were pressure control of 10 cm H_2_O, with a set rate of 10 breaths per minute and 10 cm H_2_O of positive end-expiratory pressure (PEEP). Hemoglobin remained stable at 79 g/L (normal range, 140-180 g/L), white blood cells 14.0 × 10^9^/L (normal range, 4.0-10.0 × 10^9^/L), and platelets 104 x 10^9^/L (normal range, 150-400 x 10^9^/L). The differential white blood cell counts were as follows: neutrophils 11.1 × 10^9^/L (normal range, 1.5-6.5 × 10^9^/L), lymphocytes 1.5 × 10^9^/L (normal range, 1.2-3.4 × 10^9^/L), and monocytes 1.3 × 10^9^/L (normal range, 0.2-0.8 × 10^9^/L). Anti-Factor Xa levels were measured at 0.49 units/mL (therapeutic range, 0.30-0.70 units/mL). Head and neck radiographs were ordered (Figure 1). Anaphylaxis, rashes, tracheostomy complications, subcutaneous emphysema, and angioedema were ruled out after examination of the patient and reviewing the chart for any new medications. Point-of-care ultrasonography demonstrated normal right and left ventricular function and a small non-distended left internal jugular vein without the presence of thrombosis.

**Figure 2:**
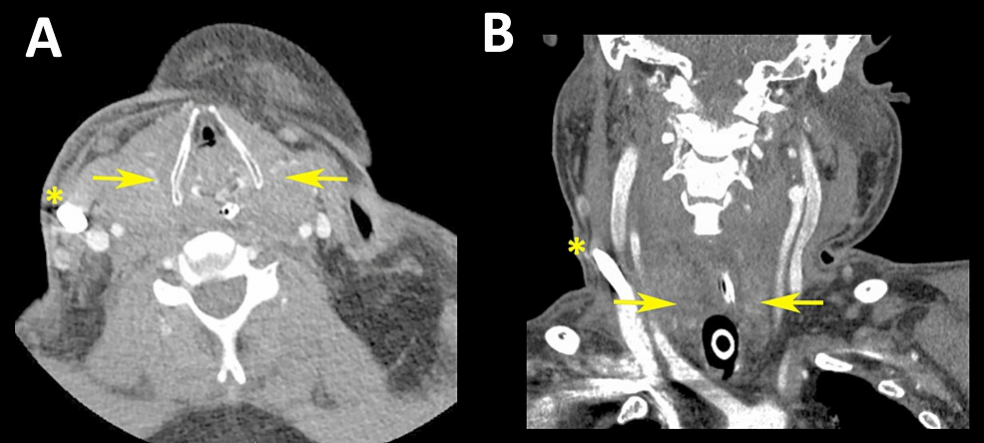
Contrast-enhanced computed tomography images of the neck. Axial view (A) and coronal view (B). The yellow arrows denote the enlargement of the thyroid. The yellow asterisk denotes the position of the extracorporeal membrane oxygenation catheter.

Potential complications were suspected and a computed tomography (CT) study with intravenous contrast was performed on the neck and soft tissues, including CT angiography and venography. The CT scan ruled out SVC thrombosis and obstruction, but it confirmed the presence of global enlargement of the entire thyroid gland (Figures 2A, 2B). A thyroid-stimulating hormone (TSH) test was performed shortly thereafter with a level of 0.23 mIU/L (normal range, 0.49-4.67 mIU/L), a free T4 of 14.8 pmol/L (normal range, 9.0-19.0 pmol/L), and a free T3 measured of 2.4 pmol/L (normal range, 2.6-5.7 pmol/L). Earlier on admission, a TSH level was measured at 0.07 mIU/L. C1 esterase levels were deferred given the CT scan findings. Overall, the clinical presentation may have been consistent with resolving subacute thyroiditis.

With conservative management, including elevating the head of the bed and ongoing use of dexamethasone, the neck swelling in our patient improved. Eventually, he was decannulated from VV-ECMO on hospital day 63, and he was discharged from the hospital after 101 days in the hospital. Follow-up lab work performed two months after hospital discharge demonstrated a normalized TSH at 1.12 mIU/L.

## Discussion

Our case highlights the unusual presentation of COVID-19-related subacute thyroiditis, resulting in acute neck swelling during VV-ECMO. This case raises a few discussion points, namely, the differential diagnosis of neck swelling, the etiology and management of subacute thyroiditis, and potential considerations for VV-ECMO management.

The incidence of severe neck and facial swelling during ECMO has not been well-described, and it has been described only in case reports [1,2]. Of these cases, they describe the onset of SVC syndrome resulting from venous obstruction due to the ECMO catheters. Due to the inherent risk of major sequelae from SVC syndrome, including cerebral edema, during ECMO, this etiology would need to be ruled out as soon as possible [5]. However, as demonstrated in our case, other etiologies for neck swelling in ECMO are also possible (Table 1). While considering the differential, we did consider the possibility that this may be COVID-19-related. Sudden lateral idiopathic neck swelling has previously been described in a case of a patient with COVID-19 after three weeks with diffuse soft-tissue swelling and edema [6]. However, our case presented with diffuse swelling rather than localized swelling. Additionally, the degree of swelling present in our case was more prominent. Hence, we explored other investigations including a CT scan which confirmed the presence of thyroiditis.

**Table 1:** Potential etiologies of neck swelling during venovenous extracorporeal membrane oxygenation.

Potential etiologies of neck swelling during venovenous extracorporeal membrane oxygenation
Superior vena cava obstruction
Facial edema
Hematoma
Sialadenitis
Thyroiditis
Angioedema
Anaphylaxis
Subcutaneous emphysema

In patients with thyroiditis, Hashimoto’s thyroiditis has been reported to be the most common cause [7]. Subacute thyroiditis is a common post-infectious complication that may arise and present with neck pain and goiter [7]. It is generally a transient condition that may manifest with transient thyrotoxicosis and suppressed TSH. COVID-19 has been increasingly recognized as an emerging risk factor for the development of subacute thyroiditis [8]. Unfortunately, as our patient had been mechanically ventilated and heavily sedated, he was unable to express any symptoms pertaining to neck pain or thyroid tenderness. Interestingly, in a systematic review of subacute thyroiditis associated with COVID-19, most cases were not identified early due to the absence of classic symptoms [8]. Many patients with COVID-19 may present with tachycardia and fever, which may mask the detection of this complication. Most cases of subacute thyroiditis respond to glucocorticoid treatment and antithyroid medications are usually not necessary [8]. In some cases of thyroiditis, patients may develop severe tachycardia and cardiovascular instability, and they may require the use of beta blockers [7].

During VV-ECMO, significant increases in a patient’s cardiac output, due to tachycardia and sympathetic activity, could result in persistent hypoxemia while on ECMO due to an increase in the shunt fraction. Fortunately, our patient did not have significant thyrotoxicosis, and he improved with conservative measures as he was already on glucocorticoids. For some patients with significant thyrotoxicosis during ECMO, some additional considerations may include watching for the development of cardiovascular collapse and cardiomyopathy [7].

## Conclusions

We report on an unusual case of acute neck swelling that developed during VV-ECMO, secondary to subacute thyroiditis from underlying COVID-19 infection. Our report highlights the importance of considering the differential diagnosis of neck swelling and investigating other etiologies, as they may potentially impact VV-ECMO management.
